# Inter-organ communication involved in metabolic regulation at the whole-body level

**DOI:** 10.1186/s41232-023-00306-1

**Published:** 2023-12-12

**Authors:** Hideki Katagiri

**Affiliations:** Department of Metabolism and Diabetes, Tohoku University Graduate School of Medicine, 2-1 Seiryo, Aoba, Sendai, Miyagi 980-8575 Japan

**Keywords:** Inter-organ communication, Neuronal signals, Metabolic homeostasis, Pancreatic β cells

## Abstract

Metabolism in each organ of multi-organ organisms, including humans, is regulated in a coordinated manner to dynamically maintain whole-body homeostasis. Metabolic information exchange among organs/tissues, i.e., inter-organ communication, which is necessary for this purpose, has been a subject of ongoing research. In particular, it has become clear that metabolism of energy, glucose, lipids, and amino acids is dynamically regulated at the whole-body level mediated by the nervous system, including afferent, central, and efferent nerves. These findings imply that the central nervous system obtains metabolic information from peripheral organs at all times and sends signals selectively to peripheral organs/tissues to maintain metabolic homeostasis, and that the liver plays an important role in sensing and transmitting information on the metabolic status of the body. Furthermore, the utilization of these endogenous mechanisms is expected to lead to the development of novel preventive/curative therapies for metabolic diseases such as diabetes and obesity.

(This is a summarized version of the subject matter presented at Symposium 7 presented at the 43rd Annual Meeting of the Japanese Society of Inflammation and Regeneration.)

## Introduction

Metabolism in each organ of multi-organ organisms, including humans, is not carried out independently, but rather in a coordinated and regulated manner throughout the body, to maintain dynamic homeostasis in response to environmental alterations. Taking glucose metabolism as an example, humans are thought to have an extremely elaborate mechanism of ensuring metabolic homeostasis with minimal elevation of blood glucose levels, even if carbohydrate intake exceeds by several dozen times the amount of glucose in the blood at each meal. However, the mechanism of this tight control remains far from being fully understood [1]. In addition to the roles of humoral factors, we have proposed the importance of inter-organ networks, i.e., the exchange of metabolic information among organs/tissues, as a mechanism(s) to maintain metabolic homeostasis at the whole-body level [2]. We have shown that the nervous system, in particular, mediates the dynamic regulation of metabolism of energy, glucose, lipids, and amino acids at the whole-body level. Herein, we present examples of inter-organ networks we have identified and discuss the significance of regulatory mechanisms based upon them, the unique roles of each organ that are being newly elucidated, the pathogenic processes underlying metabolic diseases, such as diabetes, obesity, and hypertension as well as the metabolic syndrome, and novel potential clinical applications to these diseases.

## Strategies for discovery of the inter-organ communication system

To elucidate the existence and mechanism(s) of metabolic information exchange among organs/tissues, we have applied the following approach. We abruptly alter metabolism in one organ/tissue of mice and examine the metabolic changes that occur in each of the other organs/tissues. If changes are detected in other organs, we proceed with intervention experiments to elucidate the mechanism(s) linking those organs. The phenomena that occur in other organs/tissues, when metabolism is altered in one organ, are assumed to be the body’s response that allows to maintenance of homeostasis in the individual. These are the key observations revealing the mechanism(s) linking the organs. The elucidation of a new mechanism for maintaining a normal metabolic state in an individual is thus the overarching goal. In addition, mimicking, in the first organ, the metabolic changes that occur in obesity allows the creation of loss-of-function models under obesity conditions, which are expected to lead to elucidation of the physiological and pathological significance of this phenomenon.

**Fig. 1:**
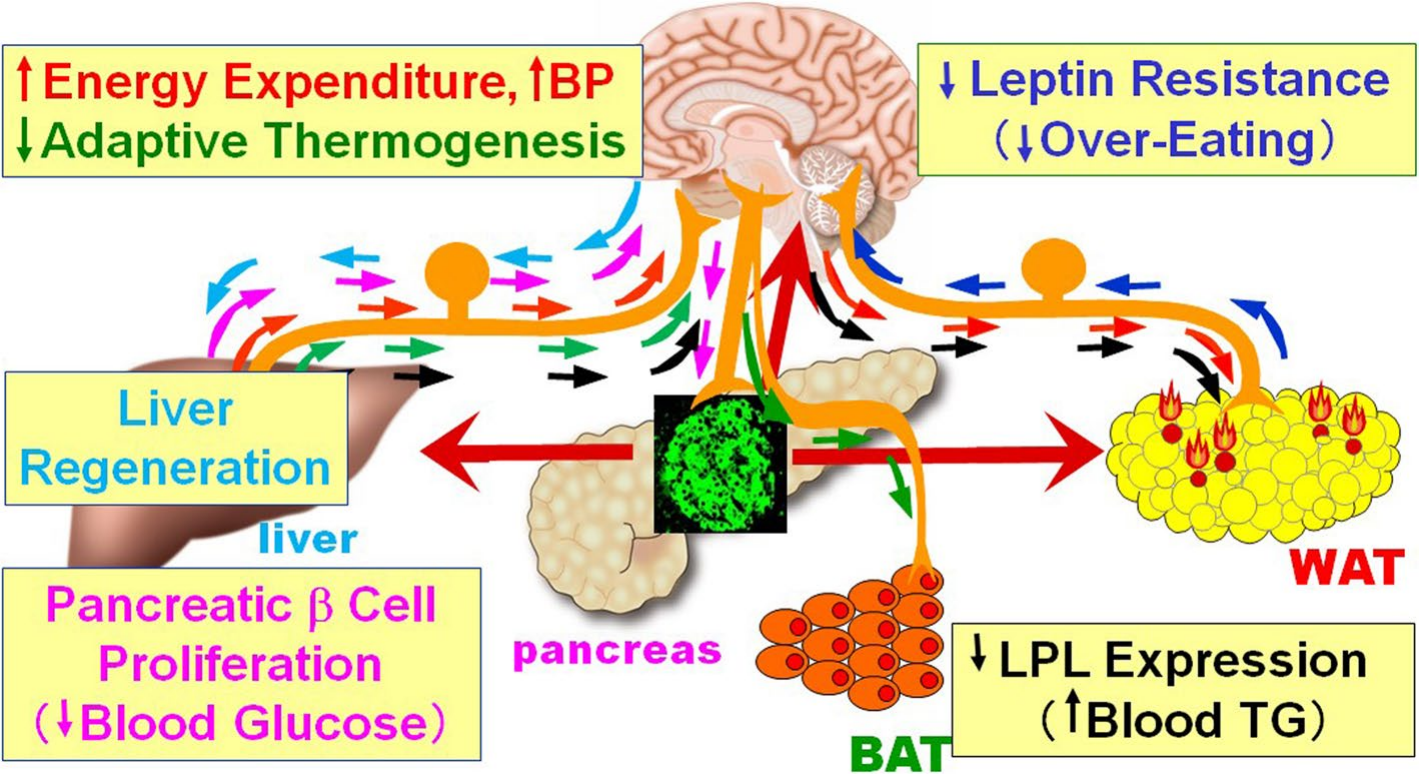
Overview of the inter-organ network mediated by neuronal signaling. WAT white adipose tissue, BAT brown adipose tissue

First, a specific example from an early study is given. Peroxisome proliferator-activated receptor (PPAR)-γ is a transcription factor that is mainly expressed in adipose tissue and induces the expression of molecules involved in lipid uptake and lipid synthesis [3]. In addition, PPARγ is also known to be upregulated in the liver in conditions such as fatty liver due to obesity [4]. Therefore, to induce fat accumulation in the liver, we hepatically expressed PPARγ using an adenoviral gene expression system [5]. As expected, PPARγ gene transfer to the liver resulted in fatty liver. Notably, lipolysis was induced in peripheral adipocytes and basal metabolism was enhanced at the whole-body level. In other words, fat accumulation in the liver triggered metabolism in other organs that favored fat burning. Various experiments were conducted to elucidate the inter-organ mechanism originating from the liver. First, we examined humoral factors, such as those secreted by the liver, but none led to this phenotype. We finally focused on neural signals. Interestingly, transection and pharmacological blockade of the vagus nerve from the liver to the central nervous system abolished all metabolism-related phenotypes in other tissues. Thus, information about excessive energy storage in the liver was transmitted to the central nervous system via the vagal afferents, leading to increased lipolysis and basal metabolism by increasing efferent sympathetic signals (Fig. 1). Furthermore, blocking the neural signals in obese states exacerbated the obesity, indicating this newly-identified neuronally mediated inter-organ communication to be a mechanism maintaining dynamic homeostasis at the whole-body level which functions to prevent obesity by enhancing energy metabolism during overnutrition [5].

## Discoveries of various types of inter-organ neuronal communication and the important role of the brain

Our next question was whether the concept of neuronally mediated inter-organ communication is limited to hepatic PPARγ-induced regulation of energy metabolism or is common to various metabolic regulatory processes. Applying similar approaches, therefore, we disturbed a variety of metabolism in a single organ/tissue and found distinct neurally mediated inter-organ communications. Adenoviral expression of uncoupling protein-1 in adipose tissue revealed that afferent nerve signals from adipose tissue regulate hypothalamic leptin sensitivity [6]. In addition, in response to alterations of hepatic metabolism, various inter-organ signals were elucidated to be transmitted, such as pancreatic β cell proliferation triggered by hepatic activation of the extracellular signal-regulated kinase (ERK) pathway [7], suppression of blood lipolysis by increased hepatic metabolism of amino acids [8], and suppression of energy consumption from increased glucose metabolism in the liver [9] (Fig. 1). These inter-organ communications are not only involved in the maintenance of metabolic homeostasis at the whole-body level but also blood pressure elevation [10], hyperinsulinemia [7], and elevated blood triglycerides [8] in obesity, as well as in the susceptibility to obesity itself [9], all of which are involved in the pathogenesis of metabolic syndrome.

Thus, information on the metabolism of each of these nutrients is transmitted throughout the body via nerves. Compared to blood-mediated humoral factor signals such as hormones, nerve signals are characterized by always being relayed through the brain [2]. Furthermore, these inter-organ communications are more organ/tissue-selective than we had initially anticipated. For example, the system that leads to an increase in pancreatic β cells is extremely selective in conveying information to the pancreatic islets of Langerhans [7]. Also, the mechanism that inhibits energy expenditure, which is initiated by increased glucose metabolism in the liver, is selective for the inhibition of heat production occurring in brown adipose tissue [9]. Thus, despite being conveyed by the autonomic nervous system, metabolic information seems to be sent with pinpoint accuracy to the target organ/tissue. In other words, it could reasonably be assumed that the brain constantly obtains information on the metabolism of peripheral organs/tissues via inter-organ communications including those transmitted by nerves, integrates the information, and regulates the metabolism of peripheral organs/tissues in a powerful and organ/tissue-selective manner to maintain optimal conditions at the whole-body level [11]. On the other hand, however, our observations could also lead to the notion that these systems, which are widely assumed to function to maintain metabolic homeostasis, are ironically involved in obesity-related pathological conditions in this era of food saturation.

## Novel inter-organ communication mediated by a humoral factor

A non-neuronal mechanism has also led to the identification of unexpected pathogenic mechanisms of obesity-related complications, such as increased gallstone formation due to an enhanced hepatic hypoxic response [12]. Humoral factors involved in pancreatic β-cell function and volume were also identified. Among them, an inflammatory cytokine, interleukin-6 [13], as well as fatty acids [14] were found to enhance glucose-simulated insulin secretion (GSIS). Additionally, microRNAs contained in exosomes secreted by bone marrow cells function to promote the proliferation of pancreatic β-cells by suppressing cell cycle inhibitors [15].

**Fig. 2:**
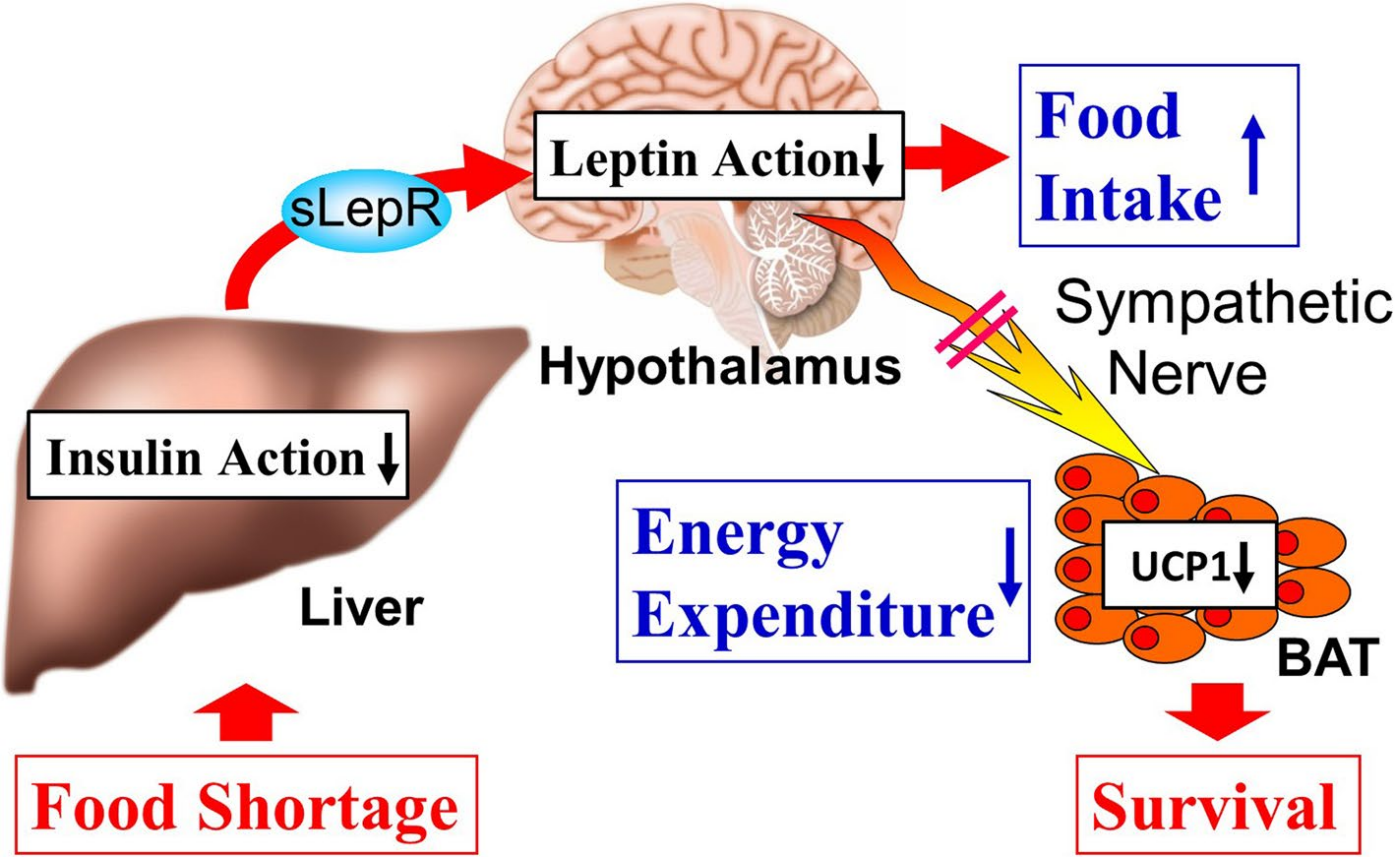
Newly discovered inter-organ network linking insulin and leptin signaling: its life-supporting function during food deprivation. UCP1 uncoupling protein-1, BAT brown adipose tissue

More recently, the mechanism by which energy expenditure is suppressed during starvation was elucidated [16]. The starting point for this study was to determine what happens when insulin signaling in the liver is suppressed by applying the aforementioned strategies. To suppress hepatic insulin signaling, we generated inducible liver-selective insulin receptor-deficient mice and obtained a weight-gain phenotype with increased food intake and decreased oxygen consumption. The mechanism of the increased appetite and decreased energy expenditure was found to be that the liver secretes soluble leptin receptors [17] (sLepRs: leptin receptors without the transmembrane domain), which bind to leptin in the blood and inhibit the action of leptin, including that in the hypothalamus, resulting in increased appetite and decreased energy expenditure [16]. This mechanism was demonstrated by the phenotype being abolished when the leptin receptor gene in the liver was additionally deleted. We then focused on the physiological significance of this organ-coupled system. Hepatic insulin signaling is well known to be suppressed during starvation. When mice were reared under restrictive feeding conditions, sLepRs in the blood were actually increased, suppressing leptin signaling in the hypothalamus and decreasing energy consumption. Importantly, when leptin receptors in the liver were deleted, the survival rates of the knockout mice decreased during restrictive feeding, which did not affect the survival rates of wild-type mice. These findings indicate that the sLepR-mediated mechanism plays an important life-supporting role under food shortage conditions [16] (Fig. 2).

## Novel role of the liver in sensing and sending metabolic information

**Fig. 3:**
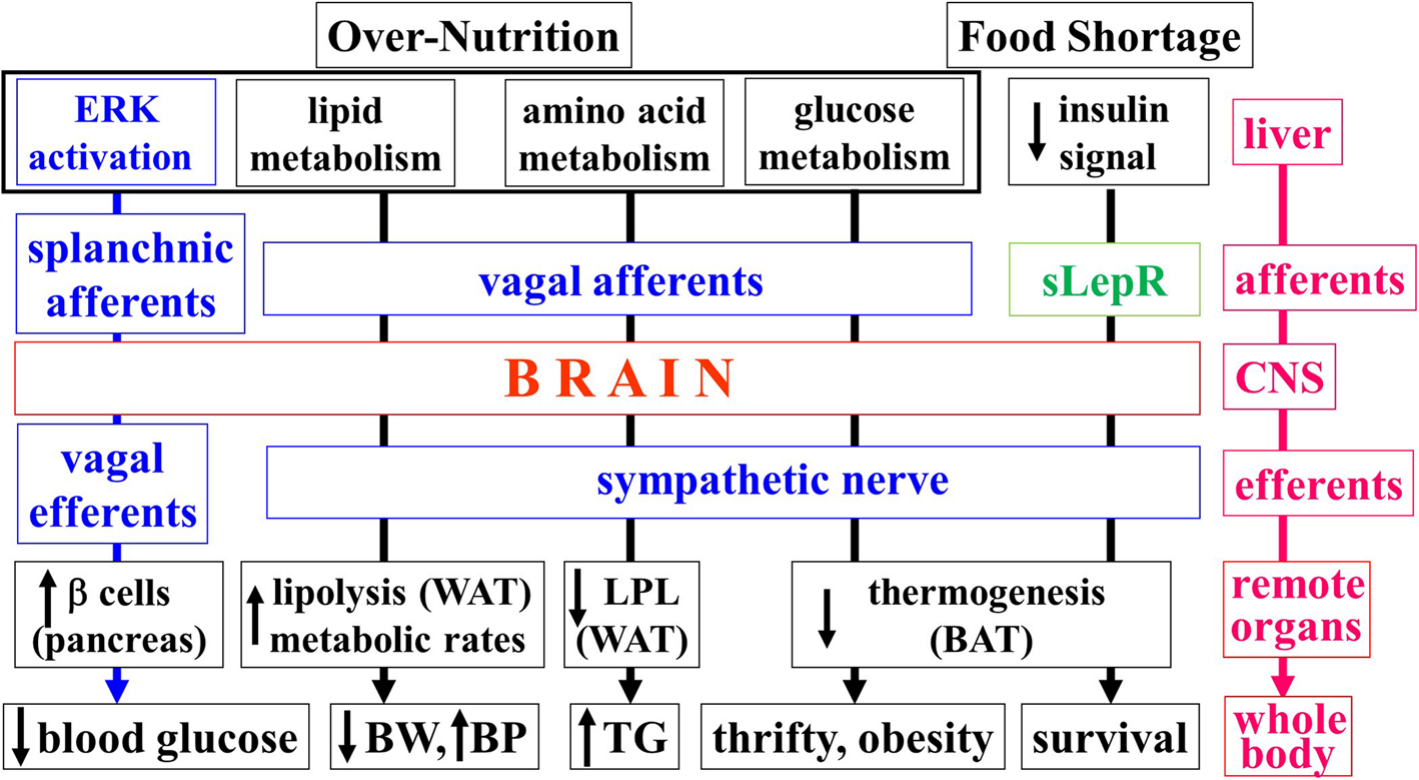
Inter-organ communications originating in the liver. The liver transmits information on metabolism of various nutrients via neuronal and humoral signals. ERK extracellular signal-regulated kinase, sLepR soluble leptin receptor, WAT white adipose tissue, BAT brown adipose tissue, BW body weight, BP blood pressure, TG triglycerides

Collectively, these research results indicate that the liver senses the metabolic state of nutrients, such as carbohydrates, lipids, and amino acids, under conditions ranging from overnutrition to starvation and sends the information on metabolic status to the central nervous system via nerve signals and humoral factors (Fig. 3). Thus, the liver seems to be always sending information, despite the liver often being called a “silent organ.” Anatomically, the liver is located such that food absorbed from the intestinal tract, cytokines associated with intestinal bacteria, and insulin and glucagon from the pancreas flow directly into it via the portal vein. Biochemically, hepatocytes themselves are a rare and distinct cell type that can store large amounts of both glycogen and fat. Given these characteristics of the liver, it is reasonable that this organ constantly senses the nutritional, as well as the immune and inflammatory, status of the intestinal tract, and the external environment, at the forefront of whole-body homeostatic control, and transmits a variety of information from the periphery. This novel role of the liver apparently makes a major contribution to dynamic homeostasis at the whole-body level in response to environmental alterations.

## Inter-organ communication leading to pancreatic β-cell proliferation

Thus, inter-organ communication systems play important roles in a variety of metabolic regulations at the whole-body level and the pathogenesis of metabolic disorders. As our next goal, we applied these mechanisms to developing therapeutic strategies of metabolic diseases, such as diabetes [18]. In doing so, a prime target among the aforementioned inter-organ mechanisms is that favoring pancreatic β-cell proliferation [7], because β cell increments may completely cure insulin-deficient diabetes. In addition, β cell function and volumes are reportedly impaired in patients with not only type 1 but also type 2 diabetes [19]. Therefore, we next endeavored to enhance our understanding of this critical inter-organ communication mechanism.

The discovery of this mechanism led us to first examine what happens in other organs when the ERK pathway in the liver is activated, which occurs in the obese states. Using an adenoviral gene transfer system, an active mutant of MAP/ERK kinase, which phosphorylates ERK, was expressed in the liver. Interestingly, hepatic ERK activation enhanced GSIS and induced acute proliferation of pancreatic β-cells. Pharmacological blockade of afferent splanchnic nerve signals from the liver, midbrain transection in the brain, and dissection of the vagal nerve innervating the pancreas each abolished the phenotype in the pancreas, suggesting that the neural network, consisting of afferent, central and efferent nerves, intervenes in this mechanism [7].

Insulin resistance occurs during obesity, which is countered by the proliferation of pancreatic β-cells, which produce and secrete more insulin. This mechanism is considered to thereby prevent the onset of diabetes. Therefore, this inter-organ neuronal network is the mechanism actually underlying the prevention of diabetes [20].

## Anatomical and molecular mechanisms leading to pancreatic β cell proliferation

**Fig. 4:**
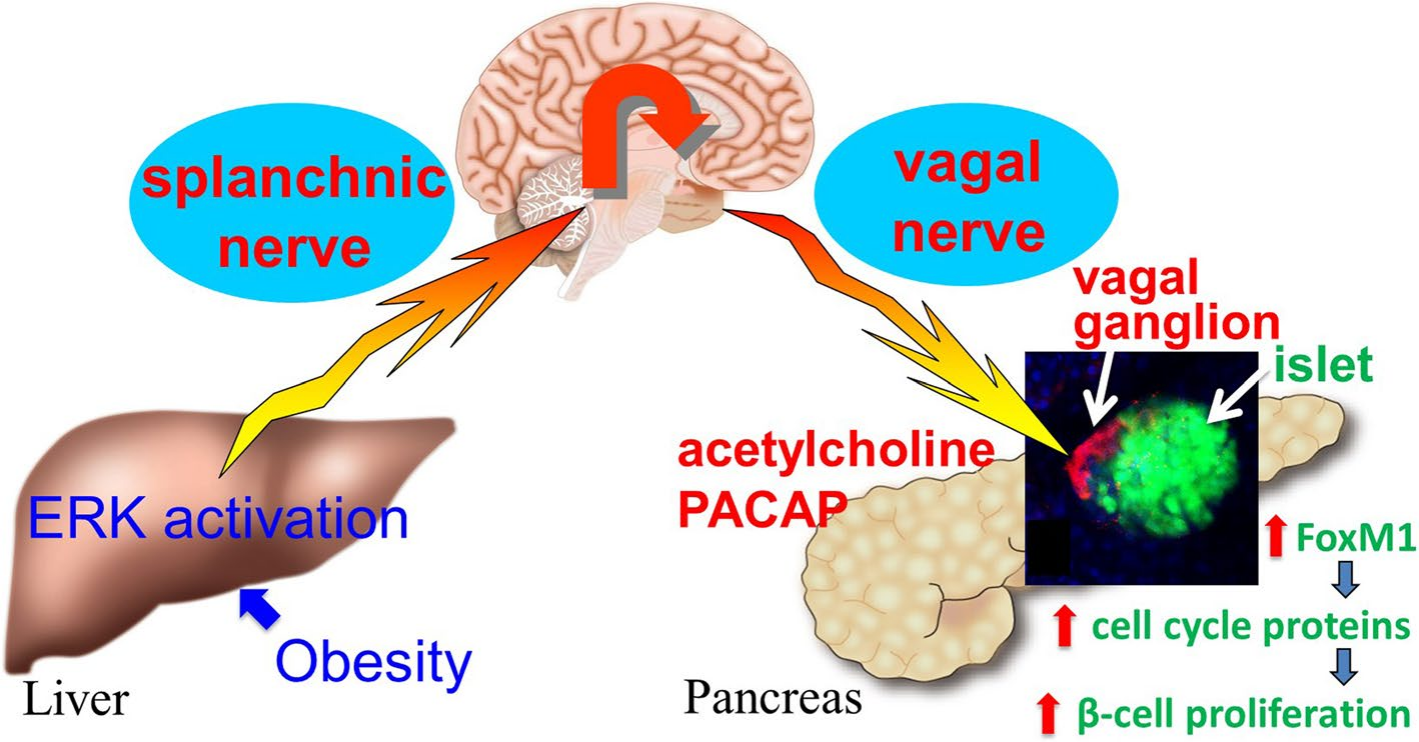
Inter-organ network leading to pancreatic β-cell proliferation. ERK extracellular signal-regulated kinase, PACAP pituitary adenylate cyclase-activating polypeptide, FoxM1 forkhead box protein M1

What is the mechanism by which information is selectively transmitted to the pancreatic islets of Langerhans, and what is the molecular mechanism by which pancreatic β-cells, which should have terminally differentiated, proliferate? It is generally known that vagus nerves project directly from the brain to various organs, including the pancreas, and form synapses with postganglionic nerves (secondary neurons) at the vagal ganglia within the organs [21]. Therefore, we made the pancreas transparent [22] and examined the localization of vagal ganglia within the pancreas, thereby revealing that the majority of intrapancreatic vagal ganglia exist in contact with the islets of Langerhans, and postganglionic nerves from their project selectively into the islets of Langerhans [23] (Fig. 4). Thus, the strong tissue-selectivity of neuronal signaling is based on this anatomical architecture. Thereafter, similar neuronal architecture was also reported in the human pancreas [24].

Postganglionic nerves are known to produce and secrete not only acetylcholine but also various neuropeptides, including pituitary adenylate cyclase-activating polypeptide (PACAP) [25]. The molecular mechanism in pancreatic β-cells leading to their proliferation was found to involve signals of both acetylcholine and PACAP secreted from the postganglionic vagus nerve. These neurotransmitters together result in activation of the β-cell Forkhead box protein M1 (FoxM1) pathway [26], thereby promoting the cell cycle and cell proliferation in β-cells [23]. In addition, β-cell FoxM1 activity underlies the maintenance of β-cell mass during adulthood, thereby preventing the impairment of glucose tolerance with advancing age [27]. Thus, via this neuronal mechanism, terminally differentiated β-cells proliferate. This mechanism functions because the nerves can create an environment in which the ligands of the two pathways (acetylcholine and PACAP) are simultaneously at high concentrations selectively in the local vicinity of β-cells in the islets of Langerhans (Fig. 4). This illustrates another major implication of the inter-organ communication mediated by neural signaling [23].

## Vagal nerve signals leading to post-injury liver regeneration

Hepatocytes are also known to have proliferative capacity [28]. Especially after injury or partial resection, these cells proliferate very rapidly to regain liver function and maintain survival [29]. We have shown that activation of the intracellular FoxM1 pathway in hepatocytes, resulting from vagal signals, is also involved in hepatocyte proliferation [30]. However, unlike pancreatic β-cells, the direct target of the neural signal is not hepatocytes, but rather macrophages, the Kupffer cells, which are also involved in this process. It is known that innervation to the liver is far sparser than that to the pancreatic islets of Langerhans and is restricted to the portal system. Thus, nerve-derived signals are considered to be amplified by Kupffer cells in the sinusoids and transmitted to the entire liver via secretion of inflammatory cytokines, such as IL-6, thereby achieving rapid and massive cell proliferation. In any case, the involvement of the vagal system in cellular proliferation to adapt and respond to various environmental alterations, ultimately leading to activation of the FoxM1 pathway in parenchymal cells, is a common mechanism that functions in both the pancreas and the liver [31].

## Successful β cell proliferation by selective stimulation of vagal nerve innervating the pancreas

As noted, previous studies mainly demonstrated the inter-organ pathways by showing the disappearance of the phenotypes when neuronal signals were blocked. In other words, although the inter-organ network system was shown to be a necessary condition, it was unclear whether the phenotype is caused by its stimulation alone, i.e., whether proliferation of pancreatic β-cells is inducible simply by activation of the vagus nerve. Therefore, we investigated whether selective stimulation of vagal nerves innervating the pancreas induces pancreatic β-cell proliferation. To achieve this aim, we combined the principles of optogenetics, material chemistry, and physics by implanting lanthanide particles, which emit blue light by upconversion in response to transmissive near-infrared light [32, 33], into the pancreases of mice expressing channel rhodopsin in cholinergic nerves [34]. By applying this technique, they succeeded in selectively stimulating the vagus nerve innervating the pancreas at a chosen timing. As a result, an increase in pancreatic β-cell function (enhancement of GSIS) was observed acutely upon stimulation, and a marked increase in pancreatic β-cells was observed chronically (intermittent stimulation for 2 weeks). Thus, activation of vagus nerves innervating the pancreas is not only necessary but also sufficient for both qualitative and quantitative enhancement of pancreatic β-cells [34].

## Attempts to increase β-cell volume by utilizing the inter-organ communication system

Thus, the inter-organ network system is a promising target for the development of preventive and curative treatment strategies for diabetes, based on both improving the function and increasing the number of pancreatic β-cells. However, it is difficult to develop a method to selectively stimulate vagus nerves innervating the pancreas and adapt it to all numerous patients with diabetes. Therefore, the elucidation of the molecular mechanism that acts on afferent splanchnic nerves from the liver is important for achieving the overarching goal of developing a curative treatment. In order to advance the research aimed at elucidation of this molecular mechanism, it was required to establish a system to monitor the proliferation of pancreatic β-cells in the same individuals over time in living mice. Therefore, we constructed a mouse system in which a secretory type of luciferase, Gaussia princeps luciferase [35], is released into the blood when the target cell type proliferates. Taking advantage of this strategy, we have succeeded in evaluating pancreatic β-cell proliferation by measuring luciferase activity in small amounts of sampled blood [36]. Depending on the Cre mice with which the model is crossed, the proliferation of other cell types can also be evaluated, making this mouse system applicable to research in a variety of fields.

## Conclusion

Many examples of inter-organ networks have been discovered as mechanisms functioning in the regulation of metabolism for various nutrients and in different nutritional states ranging from overnutrition to starvation. In addition, incidentally, their involvements in the pathogenesis of features of the metabolic syndrome, such as hypertension, hyperinsulinemia, dyslipidemia, and obesity itself have also been clarified. Furthermore, novel roles of organs have been elucidated; e.g., the brain functions as a metabolic center, and the liver senses and transmits metabolic status. The autonomic nervous system sends specific signals to target organs/tissues selectively. Taking advantage of these elaborate inter-organ mechanisms, multi-organ organisms dynamically maintain metabolic homeostasis in response to environmental alterations.

Manipulating these inter-organ mechanisms as a means to developing novel therapeutic strategies, which may even enable individuals to prevent/cure metabolic disorders, is attracting growing research attention. Diseases such as diabetes may actually be curable since β cell function and volumes are reportedly impaired in patients with not only type 1 but also type 2 diabetes [19]. Utilization of the body's endogenous inter-organ network is anticipated to induce regeneration of the patient’s own β-cells at sites where these cells should be. On the other hand, elucidating the mechanism by which the number of cells is reduced is also important [37]. Additional many issues remain to be clarified. As mentioned earlier, the molecular mechanism of afferent nerve activation is an important subject of research. Unlike the mechanism of action of the efferent autonomic nervous system, that underlying afferent nerve activation is an important field that has yet to be sufficiently addressed in the area of life sciences overall. Thus, advances in this research are expected to lead to a further understanding of homeo-dynamic mechanism under normal conditions as well as the pathophysiology of various metabolic diseases, thereby paving the way to the development of curative strategies.

## Data Availability

Not applicable.
